# Limits of use of social media for monitoring biosecurity events

**DOI:** 10.1371/journal.pone.0172457

**Published:** 2017-02-23

**Authors:** Marijke Welvaert, Omar Al-Ghattas, Mark Cameron, Peter Caley

**Affiliations:** 1 Commonwealth Scientific and Industrial Research Organisation, Canberra, Australia; 2 Plant Biosecurity Cooperative Research Centre, Canberra, Australia; Universidad Veracruzana, MEXICO

## Abstract

Compared to applications that trigger massive information streams, like earthquakes and human disease epidemics, the data input for agricultural and environmental biosecurity events (ie. the introduction of unwanted exotic pests and pathogens), is expected to be sparse and less frequent. To investigate if Twitter data can be useful for the detection and monitoring of biosecurity events, we adopted a three-step process. First, we confirmed that sightings of two migratory species, the Bogong moth (*Agrotis infusa*) and the Common Koel (*Eudynamys scolopaceus*) are reported on Twitter. Second, we developed search queries to extract the relevant tweets for these species. The queries were based on either the taxonomic name, common name or keywords that are frequently used to describe the species (symptomatic or syndromic). Third, we validated the results using ground truth data. Our results indicate that the common name queries provided a reasonable number of tweets that were related to the ground truth data. The taxonomic query resulted in too small datasets, while the symptomatic queries resulted in large datasets, but with highly variable signal-to-noise ratios. No clear relationship was observed between the tweets from the symptomatic queries and the ground truth data. Comparing the results for the two species showed that the level of familiarity with the species plays a major role. The more familiar the species, the more stable and reliable the Twitter data. This clearly presents a problem for using social media to detect the arrival of an exotic organism of biosecurity concern for which public is unfamiliar.

## Introduction

Monitoring of human health based on internet search queries, also known as syndromic surveillance, has gained popularity over the past decade following successful case studies like Google Flu (www.google.org/flutrends/about; following the footsteps of [1]) and Flu Detector [2]. The concept of using the Internet searches to monitor for syndromes rather than clinical or laboratory diagnoses has been proven to be effective in public health monitoring, though not as effective as first thought [3]. Syndromic surveillance has also been discussed in the context of bio-terrorism (see [4] for an example). Again with the public health in mind, bio-terrorism investigators are looking for a real-time systematic collection of reliable indicators of disease in individuals. The successes of syndromic surveillance in human health offer new perspectives for biosecurity surveillance, in which detecting exotic pests and pathogens of concern to the environment and agriculture in particular are the focus. Detections of pest incursions need to be timely. For example, fast reproducing pathogens (e.g. rusts) that are easily spread either mechanically or naturally may need to be detected within days for there to be any chance of eradication. Current methods of surveillance typically rely on trapping and/or inspections, which are resource intensive and hence limited in spatial and temporal extent. It has been suggested that syndromic-type surveillance techniques applied to social media data streams could provide effective biosecurity surveillance, though this has not been tested thus far.

Biosecurity surveillance aims to protect the natural environment, plants and animals, as well as agri- and horticulture from harm caused by pests and diseases [5]. Compared to self-reported syndromic human health surveillance, detecting biosecurity events from human communications faces some particular challenges. For example, the impacts and/or symptoms of exotic pests and diseases may be unknown or hard to detect, resulting in varying levels of detectability [6]. Furthermore, the direct physical impact of exotic pests on the general public can be limited in comparison with human diseases. For example, influenza affects individuals physically whereas, for example, the combined effects of citrus greening disease (Huanglongbing) and its vector the Asian citrus psyllid (*Diaphorina citri*) are neither immediate nor direct on human individuals *per se*, until it has spread significantly and affected trees are showing visible symptoms.

Therefore, when considering monitoring for biosecurity events using social media, there are several key questions to be addressed. First, would people search for or post information about biosecurity events? Second, how reliable is that information and can we access it? Third, can we validate the social media data with ground truthed data? In this paper, we conducted a validation study to assess the potential of monitoring biosecurity events on social media. This validation study is based on tracking two species that migrate each Spring (September to November) to Canberra, Australia. The research question is whether we can infer the arrival of these species by monitoring Twitter data. The potential of Twitter for syndromic-type surveillance has been demonstrated [7], though cautionary tales and considerable skepticism remains [8].

While the two species under consideration in this paper are not considered as biosecurity threats, they act as a surrogate model for real biosecurity invasions for which no validation data are available. We are particularly interested in whether we can detect their arrival in a specific region in a timely manner.

The remainder of this paper is organised as follows: the Methods section provides details on the migratory species included in this study; the data collection procedures for the Twitter data and ground truth data; the query development steps; and the analysis techniques used. In the Results, we describe the findings for our three main research questions: (1) Are the species reported on Twitter?; (2) Can we extract the relevant tweets for these species?; and (3) Are the Twitter data related to ground truth data? Finally, the Discussion extrapolates our results to the broader implications for biosecurity surveillance using social media.

## Methods

### Description of migratory species

#### Bogong moth

**Fig 1:**
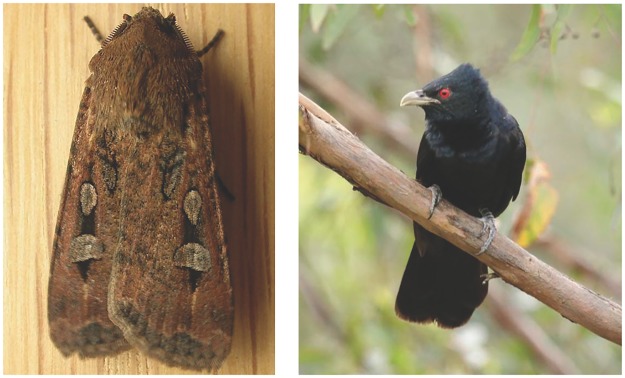
Left: Bogong Moth, Aranda, Australian Capital Territory (source: D. Hobern, www.ozanimals.com/Insect/Bogong-moth/Agrotis/infusa.html). Right: male Eastern Koel, Kobble Creek, SE Queensland (source: www.ozanimals.com/Bird/Pacific-Koel/Eudynamys/orientalis.html).

The Bogong moth (Fig 1, left), *Agrotis infusa*, has a historic tradition of migrating to the wider Canberra region. They were an important food source for the Aboriginal tribes that lived on these lands [9]. While the adult moth is not a biosecurity threat, its caterpillars, known as cutworms, are considered an agricultural pest that causes significant damage to crops. During Spring, masses of Bogong moths migrate from southern Queensland and western New South Wales to the higher altitudes of the southern part of the Great Dividing Range to aestivate over Summer. After Summer, they migrate back north and west to breed in warmer conditions on the inland plains [10].

The annual migration of the Bogong moth into Canberra is a well-known phenomenon that sometimes takes on plague proportions. Lured by the light, they are notorious for invading Canberra’s Parliament House [11]. Canberrans are familiar with the moths and have termed the migration season *#mothapocalypse* on the social network Twitter. The moth can be easily identified because of distinctive markings on the wings. In our study, the Bogong moth will serve as a surrogate model for a species that is well-known to the general public and part of a cultural phenomenom.

#### Eastern Koel

The common koels, *Eudynamys scolopaceus*, are three species of cuckoos of which the Eastern Koel, *Eudynamys orientalis*, is most regularly seen in Australia (Fig 1, right). Both common names are used interchangeably. The Eastern Koel migrates to Australia from south-east Asia to Australia to breed during Spring. After the breeding season, they return north. Koels are brood parasites (i.e. they lay their eggs in the nests of other bird species).

Up until 10 years ago, sightings of koels were very rare in Canberra, but recently Australia’s capital has been put on the migration map of the bird species [12]. One theory attributes this to the abundance of food sources in the form of ornamental plants and weeds with berries. Another factor might be the proliferation of the Red Wattlebird, *Anthochaera carunculata*, which is an effective natural host for the brood parasite [13].

Although koels are not a biosecurity threat, people often perceive them as a nuisance because of their loud, distinctive and prolonged call, often very early in the morning. This elicits a strong, polarised response in people—some absolutely hate to be woken up while others love it. Most people may not have seen a Koel but have most likely heard one at some point. Therefore, the koel forms a nice contrast to the Bogong moth, in the sense that the general public is not very familiar with sighting the bird but its presence causes high emotions (e.g. a local politician suggesting a cull to eradicate the bird species on the mistaken belief it was an exotic, introduced species, [14]).

### Data retrieval procedure

#### Twitter data

The CSIRO Emergency Situation Awareness (ESA) system is built to detect and report alerts related to crisis situations (e.g. fire, earthquake) from Twitter feeds [15]. As a side-product the system also stores tweets that can be traced back to Australia starting from 1 July 2013. Geolocation is based on either the geotag of the tweet (roughly 1% of all tweets) or the location set in the user profile (see [16] for more details). We used this database to query and extract tweets. A summary of the relevance of Twitter data is available through the Dryad digital repository (http://dx.doi.org/10.5061/dryad.7jd15).

#### Ground truth data

The Bogong moth ground truth data are the result of frequent planned surveys of the top of Mount Gingera, Australian Capital Territory, Australia, carried out by PC and MW during the period September 2014 to March 2015. Mount Gingera is well known as a preferred aestivation site with suitable boulder habitat and cool temperatures (elevation 1855m a.s.l.). Along a 1K transect, running along the summit from -35.57729 and 148.78058 to -35.57015 and 148.77405, 39 geotagged survey sites were inspected for the presence of Bogong moths. The number of moths was approximated by measuring the volume of moth patches in the crevices for each site. For each moth patch, a width, height and depth was measured. Each crevice can harbour multiple patches and each survey site has multiple crevices. The volumes of all moth patches were added for each survey site. The surveys started before the arrival of the moths and continued until their departure. We calculated the percentage of total volume for each survey and used this as ground truth data to validate the Twitter data. For the 2013-2014 migration season, no survey data are available, but the first sighting of the Bogong Moth in Canberra was reported through the media [17] and we used this date, 7 September 2013, as the anchor point for the start of the migration that season. All moth survey data are available through the Dryad digital repository (http://dx.doi.org/10.5061/dryad.7jd15).

The Koel ground truth data were obtained from the Canberra Ornithologists Group (COG), who have been running the Garden Bird Survey (GBS) since 3 July 1981. The monthly survey counts the maximum number of birds of a species seen at any time during the survey week. Survey sites are located predominantly in urban ACT at the observer’s home or workplace and are about 3Ha in area. Data are available through the Atlas of Living Australia [18]. Because the data were incomplete to do a year-by-year comparison, the monthly historical data were summed and the total percentage of sightings for each month was calculated to derive the migration season. This historical migration season is used to validate the Twitter data.

### Query development steps

To test the usefulness of social media surveillance for biosecurity events, we constructed three types of queries for each species: (1) a taxonomic query using the taxonomic classification, (2) a common name query using the common name of the species, and (3) a symptomatic query that searches for tweets that indicate the presence of the species without mentioning either the taxonomic or common name. Table 1 provides an overview of the specific queries used in this paper. Each query was used initially to retrieve tweets from the ESA system for the period July 1, 2013 to July 2, 2014.

**Table 1:** Overview of the queries used in this study. Three types of queries are distinguished: (1) a taxonomic query using the taxonomic classification, (2) a common name query using the common name of the species, and (3) a symptomatic query that searches for tweets that indicate the presence of the species without mentioning either the taxonomic or common name.

Type	Query
Taxonomic	“agrotis infusa”
Taxonomic	“eudynamys orientalis”
Taxonomic	“eudynamys scolopaeus”
Common name	(bogong OR bogan) AND moth
Common name	“common koel”
Common name	“eastern koel”
Common name	koel
Symptomatic	moth AND (first OR invasion OR plague OR season OR today)
Symptomatic	moth AND (canberra OR first OR invasion OR just OR like OR little OR one OR plague OR season OR sydney OR thought OR today)
Symptomatic	bird AND (annoying OR bloody OR love OR male OR morning) AND (call OR heard)
Symptomatic	bird AND (annoying OR bloody OR love OR male OR morning OR call OR heard)
Symptomatic	bird AND (annoying OR back OR bloody OR call OR can OR common OR going OR heard OR love OR male OR morning OR now OR one OR time)
Symptomatic	(bird OR cuckoo) AND (call OR sound OR song OR sing OR hear OR noise OR loud OR cooing) AND (“alarm clock” OR “shut up” OR morning OR annoying OR sleep OR damn OR bloody OR stfu OR night OR repeat)

To create the symptomatic query, we used the tweets resulting from the common name search as the input for a text mining analysis using the R package *tm* [19]. The goal of this analysis was to identify search terms that are commonly used to describe the sighting of the species.

The input tweets were preprocessed by (1) converting all text to lower case, (2) removing numbers, punctuation and English stop words, (3) stripping of white space and (4) stemming (i.e. removal of common word endings, for example, words ending on -s, -ed, -es) using the R package *SnowballC* [20].

The most frequent terms were identified as words that appeared at least 5 times in the enlisted tweets. For the Bogong moth, this resulted in the following 13 terms: “Canberra”, “first”, “invasion”, “just”, “like”, “little”, “moths”, “one”, “plague”, “season”, “Sydney”, “thought” and “today”. We constructed the following queries:

1. moth AND (first OR invasion OR plague OR season OR today)
2. moth AND (Canberra OR first OR invasion OR just OR like OR little OR one OR plague OR season OR Sydney OR thought OR today)

The first query is a specific one that focuses on the most frequent terms. The second query uses all the key words that came up in the frequency analysis and is expected to be more general.

For the Koel, this resulted in the following 16 terms: “annoying”, “back”, “bird”, “bloody”, “call”, “calling”, “can”, “common”, “going”, “heard”, “love”, “male”, “morning”, “now”, “one” and “time”. Based on these terms, three different symptomatic search queries were constructed:

1 bird AND (annoying OR bloody OR love OR male OR morning) AND (call OR heard)
2 bird AND (annoying OR bloody OR love OR male OR morning OR call OR heard)
3 bird AND (annoying OR back OR bloody OR call OR can OR common OR going OR heard OR love OR male OR morning OR now OR one OR time)

The first query aims to maximize the signal by combining the search terms semantically while not using the more general terms. The second query uses the same terms as query 1, but minimizes specific combinations. The third query is the more general one, using all the terms identified in the text mining analysis. It is expected that this will result in more noise. Besides the text mining analysis of the tweets, for the Koel bird, we also performed a subjective selection of search terms. These were categorized in three groups: (1) terms describing the species, (2) terms related to the sound (i.e. Koels have a specific call), and (3) terms related to time of sighting and emotions caused by the Koel. This resulted in the following query:

4 (bird OR cuckoo) AND (call OR sound OR song OR sing OR hear OR noise OR loud OR cooing) AND (‘alarm clock’ OR ‘shut up’ OR morning OR annoying OR sleep OR damn OR bloody OR stfu OR night OR repeat)

### Analysis methods

#### Signal-to-noise ratio

We used the different queries outlined in Table 1 to extract the tweets from the ESA database. Where feasible (i.e. the results were less than 1,000 tweets) we then manually processed the content of the tweets to assess their relevance. Relevance is defined here as whether the tweet related to the species of interest or not. This can be describing a sighting, reacting to media reports, assisting in identifying a sighting, etc. In the context of our surveillance exercise, only the relevant tweets are the true signal-of-interest. Therefore, the ratio of the number of relevant tweets over the number of hits will give us the signal-to-noise ratio (SNR) of the query. So, the SNR represents the proportion of the signal we actually want to capture and therefore, the higher the SNR, the better. In the case that the query result exceeded 1,000 tweets, we used a stratified sampling approach to create a subsample of the tweets while accounting for the total number of tweets per month in that query. Subsamples were created for the second Bogong moth symptomatic query, and the second and third Koel symptomatic query, comprising of 10%, 5% and 1% of the total hits for those queries respectively. Based on the subsamples, an approximate SNR was calculated as described above.

#### CUSUM analyses

In addition to a visual comparison of the Twitter data and ground truth data, we performed a CUSUM (cumulative sum) analysis as a statistical control measure of the Twitter data and to monitor change detection [21, 22]. All CUSUM charts were constructed using the R package qcc [23] based on the default values provided by the package. To account for the potential violation of the independence assumption of the CUSUM estimation due to auto-correlation in the Twitter data, we performed the CUSUM analyses on the independent residuals of an Auto Regressive (AR) model rather than the raw Twitter data [24]. For each Twitter time series, we performed the following procedure:

1. Inspect the auto-correlation function (ACF) and partial auto-correlation function (PACF) of the time series
2. Fit an AR model with parameters chosen based on the ACF and PACF plots
3. Check the model diagnostics, in particular the ACF of the residuals for independence
4. Construct a CUSUM chart of the standardised AR residuals

## Results

### Species reporting on Twitter

**Fig 2:**
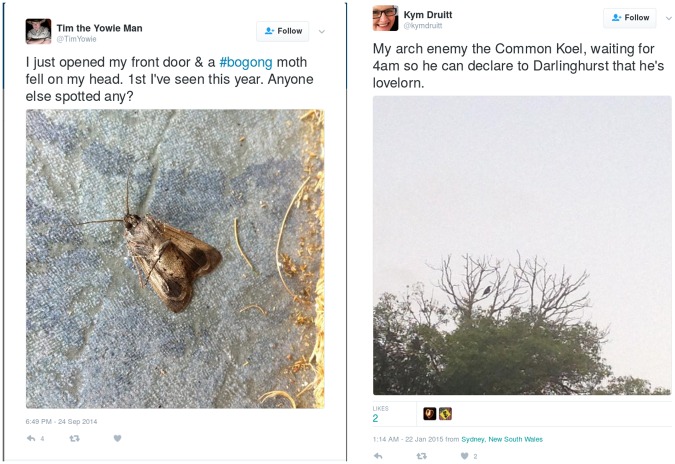
Examples of Twitter conversation on the Bogong moth (left) and the Koel (right). Left figure reprinted from Twitter under a CC BY license, with permission from Tim the Yowie Man, original copyright 2014. Right figure reprinted from Twitter under a CC BY license, with permission from Kym Druitt, original copyright 2015.

A manual search of Twitter conversations indicated that there are indeed tweets pointing to the presence of both species. Examples of these conversations are presented in Fig 2. Therefore, with respect to our first research question, we confirmed the reporting of both migratory species on Twitter. The next question is whether we can actually retrieve those tweets reliably enough to be useful as a presence signal.

### Retrieval of the relevant signal

Table 2 provides an overview of the results (i.e. number of hits, number of relevant tweets and the SNR) for the different queries described in Table 1.

**Table 2:** Number of hits from 1 July 2013 to 2 July 2014 for different queries as outlined in Table 1. The relevant hits were counted after manual processing of the tweet content (where feasible). The signal-to-noise ratio (SNR) represents the ratio of the number of relevant tweets over the number of total hits for each query.

Query type	Query	Hits	Relevant	SNR
Taxonomic	Moth	2	1	50%
	Koel 1	0	–	–
	Koel 2	0	–	–
Common name	Moth	98	88	90%
	Koel 1	22	22	100%
	Koel 2	6	6	100%
	Koel 3	409	159	39%
Symptomatic	Moth 1	520	308	59%
	Moth 2	5,629	–	47%^⋆^
	Koel 1	120	12	10%
	Koel 2	11,534	–	4%^⋆^
	Koel 3	48,016	–	2%^⋆^
	Koel 4	480	63	13%

^⋆^ Approximate SNR based on a stratisfied sample

#### Taxonomic queries

Taxonomic queries were unsuccessful for both species in that they result in no or a very limited number of hits (Table 2).

#### Common name queries

Common name queries gave us mixed results. For the Bogong moth (Query “Moth”, Table 2), this is highly successful, with 90% of the tweets being relevant. On the other hand, although the SNR is 100%, the first two queries for the koel, searching for Common Koel (Query “Koel 1”, Table 2) or Eastern Koel (Query “Koel 2”, Table 2), are less successful because of their limited number of hits. In contrast, the last query (Query “Koel 3”, Table 2) has a relatively large number of hits but the SNR is only 39%. Reasons that immediately come to mind for the lowered SNR include that koel is the Dutch and South-African word for cool, and Koel is also the name of hugely popular Indian television show, hence there are quite a few Twitter usernames that have koel in them. Therefore, it is quite possible that the SNR can be increased by further filtering of the query.

#### Symptomatic queries

By and large, the symptomatic queries resulted in the largest number of hits, however the SNR also seems to be the lowest. We could only determine the SNR for 3 of the 6 queries because the number of hits for the other queries was too big to manually process. Therefore, we will only discuss the results for the former. For the bogong moth, the first symptomatic query (Query “Moth 1”, Table 2) does relatively well with a SNR of 55%. However, the symptomatic queries for the koel (Query “Koel 1” and “Koel 2”, Table 2) deliver only 10% and 13% of relevant signal, respectively. Although the process to build the symptomatic query was the same for both species, we do get quite different results, remembering that both species are characterised by very similar spatial and temporal migration patterns. Therefore, it could be expected that the underlying tweeting population is the same.

Before moving on to the validation of the signal, we will discuss the quality of the information obtained thus far. When using monitoring techniques on social media, one has to be aware of the fact that the information extracted from this medium is not validated. For example, during the manual processing it became apparent that for some tweets we could assume that they refer to the sighting of the species, but in most cases we cannot verify whether that information is correct or not. A distinction can be made between tweets that are verifiable (i.e. it can be assessed whether it is a true positive or false positive) and tweets that are unverifiable. The information content of the tweets is generally verifiable if a link is provided to a video or photo. Unfortunately, this is only the case for a minority of the tweets. For example, for Symptomatic Query “Koel 1”, 9 out of the 12 relevant tweets were unverifiable. The three tweets that were verifiable because of enclosed photographic material turned out to be false positives (i.e. the tweet related to a different bird species).

### Validating the signal

**Fig 3:**
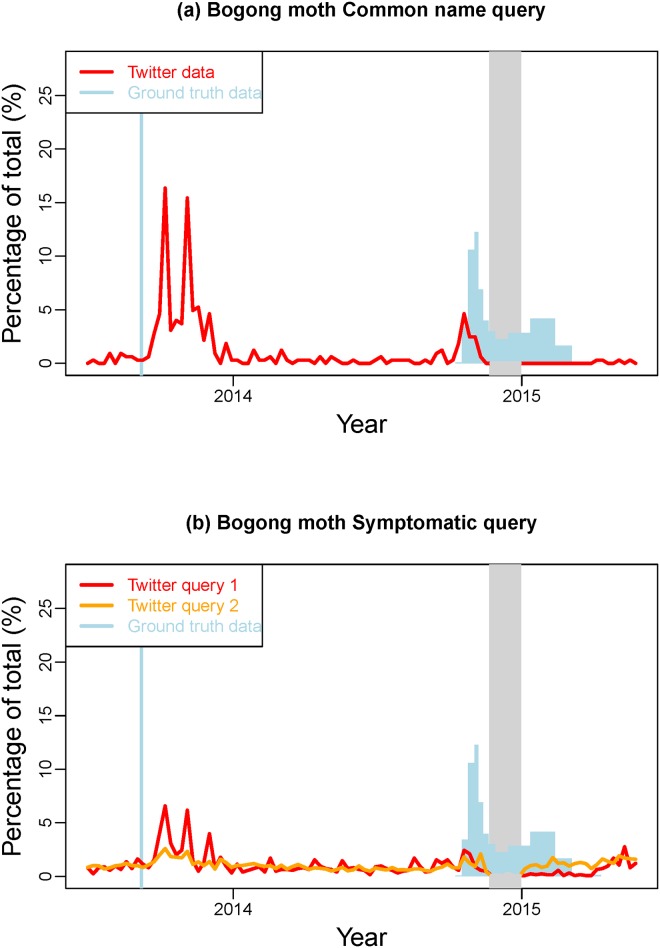
Validation of Bogong moth Twitter data against ground truth data collected in surveys. The Twitter data are represented as time series of the weekly counts, while the survey data are shown as bar charts. The grey shaded area delimits the time period in which tweets couldn’t be reliably captured.

**Fig 4:**
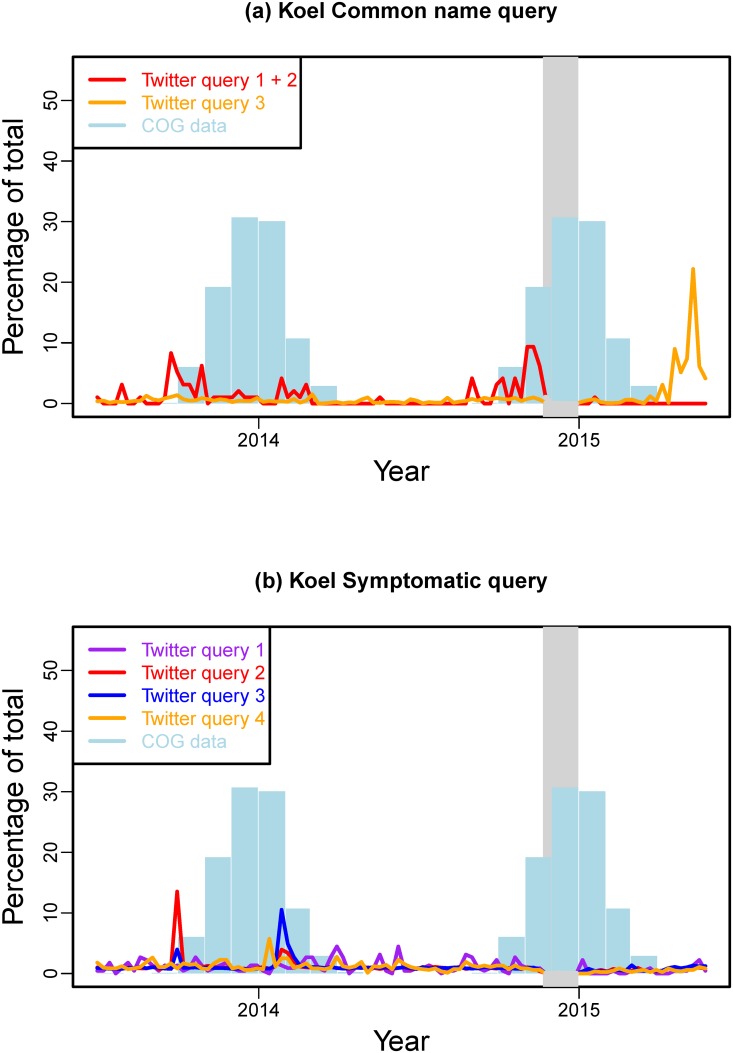
Validation of Koel Twitter data against historical monthly sightings. The Twitter data are represented as time series of the weekly counts, while the historical data are shown as monthly bars that are replicated for each migration season. The grey shaded area delimits the time period in which tweets couldn’t be reliably captured.

For the validation of the Twitter data, we extended the time frame for the retrieval of the Twitter data such that two migration seasons are captured. Figs 3 and 4 present the results for the Bogong moth and the Koel, respectively, from 1 July 2013 to 31 May 2015. By validating the Twitter data to ground truth data we want to ensure that observed peaks in the Twitter data are actually related to the phenomenon of interest (i.e. migration of the Bogong moth or Koel).

#### Visual inspection

For bogong moth, we can observe clear peaks in the common name query (Fig 3a) at the start of each season. During the 2013–2014 season, there seem to be two waves of information. Inspection of the content of the tweets reveals that this second wave is mainly due to reactions of media reports, which are reporting an unusual early and large Bogong moth migration. By comparison, the peak in the common name query for the 2014–2015 season is much smaller. This is in line with the reporting of experts [25]. Further, it appears that the Twitter data only capture the arrival (e.g. first sighting only) of the moths rather than a sustained reporting over the whole time period when the moths are in the region. This is partially explained by aestivating moths restricting their movements to the immediate vicinity of their aestivation site. Part of these results can also be observed in the data from the first symptomatic query (Fig 3b, blue line). Although decreased in size, we see a few peaks for the 2013–2014 season that are also slightly delayed compared to the common name query data. However, there is no obvious increased signal for the 2014–2015 season. The second symptomatic query (Fig 3b, green line) does not provide any evident results.

For the Koel, Fig 4a presents the common name queries results. Here, we use the COG garden survey data as a validation point for the Twitter data. To augment the signal, we combined the first two common names queries (i.e. adding up the results for the Eastern Koel and Common Koel search). This results in small increases at the beginning of each migration season. The third common name query, however, does not result in any usable data. On the contrary, there is a peak during April–May 2015 that is completely out-of-season and therefore, is not related to the arrival of migrating Koel. The symptomatic queries (Fig 4b) present an even more diffuse picture. There is no clear relationship between any of the symptomatic queries and the historical COG data. Any visible peaks can be traced back to events that are not related to the Koel migration (e.g. revival of the “Flappy bird” computer game).

#### CUSUM analysis

The ACF analyses of the Twitter time series revealed auto-correlation patterns with varying lags. Table 3 presents the exact lag parameters that were used as input to fit an AR model using the *arima* function in the statistical software package R [26]. For both the AR fitting and the CUSUM analyses we only used the combined common name query as described above. The standardised residuals of the AR models were all independent and were then used to obtain the CUSUM charts.

**Table 3:** Lag parameters used to fit AR models for the Twitter time series.

Bogong Moth	Common name	5
	Symptomatic 1	5
	Symptomatic 2	5
Koel	Common name 1+2	3
	Common name 3	4
	Symptomatic 1	4
	Symptomatic 2	1
	Symptomatic 3	3
	Symptomatic 4	5

**Fig 5:**
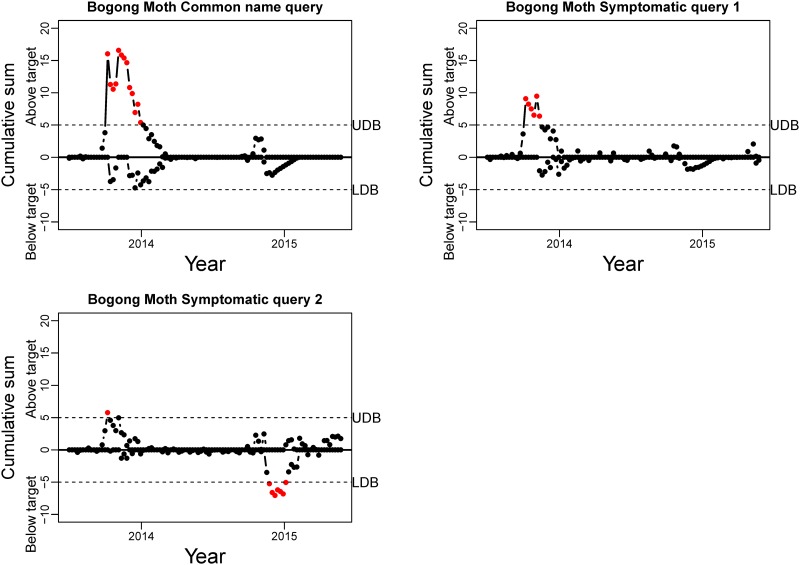
CUSUM charts for the Bogong moth queries. Red dots indicate a deviation of the number of tweets, in particular the upper part of the chart points to an increase in tweets. UDB: Upper Decision Boundary; LDB: Lower Decision Boundary.

The CUSUM charts for the standardised residuals of the Bogong moth queries are presented in Fig 5. The charts are interpreted as follows: As long as the points fall within the Upper Decision Bound (UDB) and the Lower Decision Bound (LDB) the process is considered *in control*. As soon as the points exceed these boundaries, the process is *out of control*. Within our validation context, we are specifically looking for out of control processes above the UDB during Spring (September—November) that mark the arrival of the Moths. Fig 5 shows that for the common name query we get a significant increase for the 2013 Spring Season. However, the 2014 Spring season, although still visible, is noticeably smaller and does not exceed the UDB. Similar results are obtained for Symptomatic query 1, but in this case the arrival in 2014 is hardly detectable. Symptomatic query 2, on the other hand, displays non-conclusive results. While the Bogong moth season is just detected during 2013–2014, the following year the out of control processes as detected by the CUSUM chart seem to detect a decrease rather than an increase.

**Fig 6:**
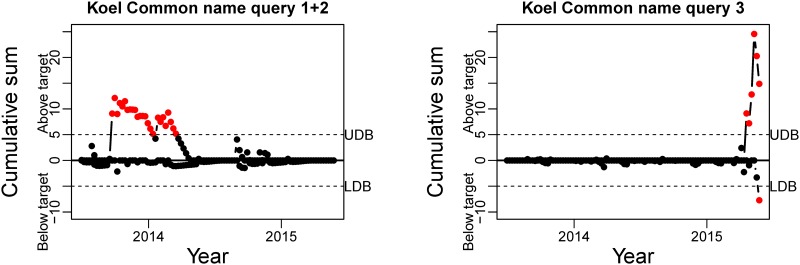
CUSUM charts for the Koel Common name queries. Red dots indicate a deviation of the number of tweets, in particular the upper part of the chart points to an increase in tweets. UDB: Upper Decision Boundary; LDB: Lower Decision Boundary.

**Fig 7:**
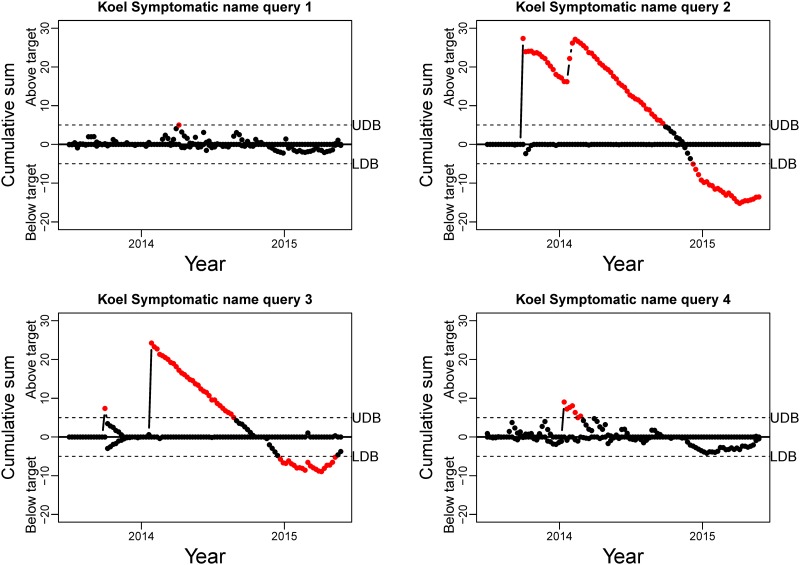
CUSUM charts for the Koel symptomatic queries. Red dots indicate a deviation of the number of tweets, in particular the upper part of the chart points to an increase in tweets. UDB: Upper Decision Boundary; LDB: Lower Decision Boundary.

The CUSUM charts for the standardised residuals of the Koel common name queries and symptomatic queries are presented in Figs 6 and 7, respectively. Similar to the Bogong Moth CUSUM charts, we are looking for points above the UDB during September–November. The results for the common name queries are not consistent (Fig 6). While the combined Common name query seems to detect the season in the first year, the second year does not exceed the threshold. Common name query 3, however, does not provide any usable results, which is consistent with our conclusions from the visual inspection. The symptomatic queries results for the Koel (Fig 7) do not display any detection that is consistent with a Spring migratory pattern. The peaks that are detected are mainly situated during the Summer of 2013-2014 and are most likely driven by the revival of the smartphone game *Flappy Bird* (as detected by manual inspection of the tweets for that time period). This is most pronounced for symptomatic query 1, while queries 2, 3, and 4 give a very diffuse pattern. Note that for these results, increasing the threshold for the UDB would not provide an improvement.

The results of this validation study, both the visual inspection and CUSUM analysis, are very much in line with the SNR results presented in Table 2. It seems that the higher the SNR for the query (i.e. the more relevant tweets compared to irrelevant tweets), the more likely that the query will provide useful information, both on timing and size, of the migration.

## Discussion

In contrast to the successful use of social media for human health surveillance and disaster detection [1, 4], similar successful applications of the using such data streams for detecting biosecurity events are lacking [27]. Machine learning algorithms are typically used to classify the tweets as pertinent or not. The underlying assumptions for these algorithms to work is that there is a defined set of distinctive features (i.e. words describing symptoms) that can be used to train the classifier on. Additionally, there are sufficient data for the classifier to be trained and tested. This paper has explored some of the challenges posed when essentially downscaling such methods to detecting biosecurity events. We note that both assumptions may not hold when trying to use the concept of syndromic surveillance for biosecurity events. First, biosecurity threats, typically being exotic/foreign, are by definition less known to the general public compared to an illness that affects them directly. Second, biosecurity surveillance relies heavily on early detection and only a limited number of data are typically available. Therefore, before attempting any machine learning approaches, we first explored the information content in the Twitter data stream to explore the possibility of using Twitter for biosecurity surveillance.

The two species we investigated migrate annually to the same region and around the same time. Differences in the history of the migration may explain the difference in their reporting rates. While the Bogong moth, has been migrating South during Spring for at least the past 10,000s years, the Common Koel, only started expanding its migration pattern more South less than a decade ago. As a consequence, there is a difference between the species in terms of familiarity amongst the general public. In particular, Canberrans are well aware of the Bogong moth migration during Spring and are very likely to be able to identify the moth by common name. They are less familiar with the Koel, however, and mainly recognise the presence of the bird based on its loud, distinctive and persistent call. The level of familiarity for both of the species is also reflected in our results. The Bogong moth common name query resulted in a higher number of hits compared to the first two Koel common name queries, while the 100% SNR for those Koel common name queries, indicates that the name is only used by knowledgeable Twitterers. The results for both species also show that only the arrival of the species can be captured in the Twitter data stream. After the initial excitement of the arrival, the attention of Twitterers most likely drifts to other more interesting topics. While this is good news in the context of early detection of biosecurity events, there are still questions on how this would apply to the detection of exotic species given the influence of the familiarity of the species on the SNR levels in the Twitter signal.

Our results clearly indicate that tweets containing taxonomic descriptions of novel species are rare. This is not unexpected, since there are few social media users that would use the taxonomic classification (i.e. experts), and probably have alternative preferred channels to report their sightings. This is in contrast with the universal recognition and interest in major events such as earthquakes, pandemics, fires etc [28]. The key difference with our biosecurity problem of early detection, is that these applications of social data mining, for example [28] are providing useful information of the spatio-temporal pattern of an event that is already detected and identified. Here, we are evaluating the use of such data streams for the detection phase. Clearly the next question relates to whether social media can usefully inform authorities on the spatial-temporal characteristics of an incursion of an exotic species. This is topic for future research in the biosecurity space, and we note the progress in spatio-temporal filtering techniques that already exist for the detection of disaster-related communication [29].

We note that half of the results for the symptomatic queries provided too many tweets to process. In that case it would be tempting to turn to an machine learning algorithm to process these tweets (see [2] for an example in human health), however, we would like to urge caution in using these algorithms before having a general idea on what the relationship is between the Twitter data and the actual event that we are interested in. Importantly, we have incorporated ground truthing as a means of validation. Whilst machine learning shows promise, we want to emphasize the important role for data interpretation and validation [3, 30].

In conclusion, when using Twitter for detecting biosecurity events, the common name approach is most likely the method with the highest change of successful surveillance. There is an intrinsic problem in such an approach—this common name surveillance only has the potential to work if the general public is sufficiently familiar with the common name of the species of interest. It is hard to imagine that the general public will have enough familiarity with exotic species during the early stages of an incursion for them to report them by common name, unless they are being sufficiently exposed to biosecurity awareness campaigns for those species. A potential solution to this familiarity problem is to use Twitter as an educative tool and by actively prompting the tweets through hashtags, active network development and dedicated Twitter accounts, rather than just passive monitoring. This could benefit biosecurity surveillance in two ways: (1) the general public gets educated around biosecurity issues, and (2) the data quality of the Twitter surveillance will increase.
